# Biomimetic CuS nanoparticles for radiosensitization with mild photothermal therapy and GSH-depletion

**DOI:** 10.3389/fonc.2022.1054608

**Published:** 2022-11-24

**Authors:** Xiaoxiang Zhou, Xiang Li, Bo Wu, Zhiran Chen, Longyun Chen

**Affiliations:** ^1^ The Yancheng School of Clinical Medicine of Nanjing Medical University, Yancheng Third People’s Hospital, Yanchen, China; ^2^ Department of Central Laboratory and Precision Medicine Center, Department of Nephrology, The Affiliated Huai’an Hospital of Xuzhou Medical University and Huai’an Second People’s Hospital, Huai’an, China

**Keywords:** radiotherapy, GSH-depletion, photothermal therapy, biomimetic, hypoxia

## Abstract

Due to its non-invasive and highly effective characteristics, radiotherapy has attracted significant interest in cancer treatment. However, radioresistance of solid tumors caused by a unique tumor microenvironment diminishes the therapeutic effect of cancer radiotherapy. To address this issue, we developed a nanoplatform for tumor-specific targeting to improve radiotherapy. Specifically, hollow CuS nanoparticles were decorated with the platelet cell membrane (PC), endowing this nanoplatform with the therapeutic property of navigating to the tumor region for glutathione (GSH)-depletion photothermal therapy. It was discovered that mild photothermal therapy mediated by PC ameliorated hypoxia in the tumor microenvironment. Meanwhile, GSH, which contributes to repairing radiotherapy-induced DNA double-strand breaks, was depleted by PC in an acidic microenvironment. Therefore, radioresistance could be diminished while cancer cell self-repair was prevented. At therapeutic doses, PC nanoparticles have negligible toxic effects on normal tissues. PC demonstrates promise for both *in vivo* and *in vitro* radiosensitization due to its GSH-depletion, photothermal efficiency, and tumor-specific properties.

## Introduction

Colon cancer remains one of the most significant global health concerns (1–3). The principal therapeutic strategies include surgery, chemotherapy, radiotherapy, immune therapy, and other methods (4, 5). Radiotherapy is typically administered after surgery or as an adjuvant therapy in conjunction with chemotherapy (4). For patients unable to tolerate surgery because of their condition, radiotherapy emerges as a viable alternative (6–8). Utilizing radiation rays to interact with the DNA of tumor cells directly or with H_2_O to produce reactive oxygen species to induce tumor cell death indirectly is the mechanism of radiotherapy. However, due to tumor radioresistance, radiotherapy for colon cancer had a limited therapeutic effect. On the other hand, increasing the radiation dose may have severe side effects on normal tissues and organs nearby (9, 10). Considering the concerns above, it is urgent to improve radiotherapy’s therapeutic effect in treating colon cancer without causing side effects.

Unlike normal cells, tumor cells frequently proliferate rapidly, differentiating tumor tissues from their normal counterparts. Tumor cells consistently outgrow their oxygen and nutrient supplies, resulting in a unique tumor microenvironment characterized by hypoxia, overexpressed H_2_O_2_, high glutathione (GSH) levels, and mild acidity (2, 11–17). Ionizing radiation causes DNA double-strand breaks (DSBs), which result in cell apoptosis by direct damage. Hypoxia-resistant tumor cells are resistant to radiotherapy. Furthermore, GSH in tumor tissues repairs DSBs induced by ionization damage, reducing radiation damage (18). Tumor cells exhibited radioresistance for these reasons, which could lead to radiotherapy failure or tumor recurrence. To enhance the effect of radiotherapy, extensive research has focused on the development of specific radiosensitizers. By photoelectric effect, radiosensitizers containing high-z elements can amplify the energy of ionizing radiation. Recent research has demonstrated that gold (19–22), bismuth (23), hafnium (24, 25), gadolinium (26), and other novel elements (27) have a remarkable radiosensitizing effect. In addition, several ground-breaking studies have revealed that microenvironment modulation may offer a novel technique for radiosensitization. For example, nanomaterials containing manganese dioxide (28, 29) or platinum (30) have enzyme-like properties that catalyze H_2_O_2_ into oxygen and may alleviate intratumor hypoxia. Other nanomaterials can reduce GSH levels. For instance, MoS_2_ nanoflowers (31) could deplete intratumoral GSH overexpression. It has been reported that combining isoniazid and core-shell magnetic nanospheres Fe_3_O_4_@MnO_2_ can effectively eliminate intracellular GSH. Thus, the radiotherapy-induced repair of GSH oxidative stress was prevented. In addition, a catalyst that converts intracellular H_2_O_2_ to ROS demonstrated a promising radiosensitization mechanism (32). Some Fe-based nanomaterials could use the Fenton reaction for this purpose (33). Other strategies increase ROS levels in different ways, including using photosensitizers to mediate photodynamic therapy, which generates large amounts of ROS (21). In additional, photothermal therapy could also enhance the effect of radiotherapy by hypoxia alleviation to obtain synergistic therapy, which was mediated by nano-radiosensitizer such as gold spikes (19). It could be concluded that designing an effective radiosensitizer to enhance the therapeutic effect is of utmost importance.

It is essential to consider the radiosensitizers’ ability to target tumors to avoid unnecessary damage to normal tissues and organs. With either passive or active targeting, nanoparticles could be used to target tumors. Due to the disordered endothelial cells and leaky vasculature of the tumor region, injected nanoparticles tend to escape from blood vessels and become retained in tumor tissues *via* these leaks. This effect is enhanced permeability and retention (EPR) (34). However, targeting efficiency is limited, and nanoparticle distribution is not uniform. Nanoparticles were decorated for active tumor targeting to combat this issue. Some nanoparticles, for instance, are conjugated to ligands, such as antibodies and molecules (35–37). Bionic technology is an emerging strategy for enhancing delivery efficiency. Recent research has focused extensively on cell membrane coatings for nanoparticle delivery (38). Red blood cells (RBC) are prevalent among blood cells, and their membrane can typically be used to coat immune-evading nanoparticles (39). In addition, platelet cell membranes, distinguished by their specific tumor targeting and immune-evasion properties, can be used to conceal radiosensitizers like Au@AuPd (20). Other cell membranes, such as those of cancer cells (38), stem cells (40), and beta cells (41) were applied to the coating for efficient targeting. The natural function of the cell membrane could be transferred to nanoparticles for targeted delivery using these biomimetic techniques.

Here, we present a platelet-cell membrane (CM)-camouflaged hollow CuS nanosphere (PC) for photothermal therapy-enhanced radiotherapy. As platelet cells tend to navigate towards inflammation by nature, one characterization of the tumor microenvironment’s PC was endowed with immune-evasion and tumor-targeting capabilities. After being administered *via* the tail vein, the PC’s relatively prolonged blood circulation time allowed it to locate the tumor site. Due to PC’s high photothermal conversion efficiency, photothermal therapy was able to kill tumor cells. Meanwhile, the elevated temperature in local tumors is attributed to accelerated intratumoral blood flow, which could bring sufficient oxygen to tumors to alleviate hypoxic status. In an acidic microenvironment, PC releases Cu^2+^, which can deplete intracellular GSH. Through this strategy, radiotherapy-induced DNA double-strand breaks could be eliminated by GSH and converted to irreversible damage by oxygen. This study demonstrated the potential of tumor-specific targeting PC for photodynamic therapy to enhance radiotherapy, opening a new avenue for antitumor therapy.

## Result and discussion

**Scheme 1:**
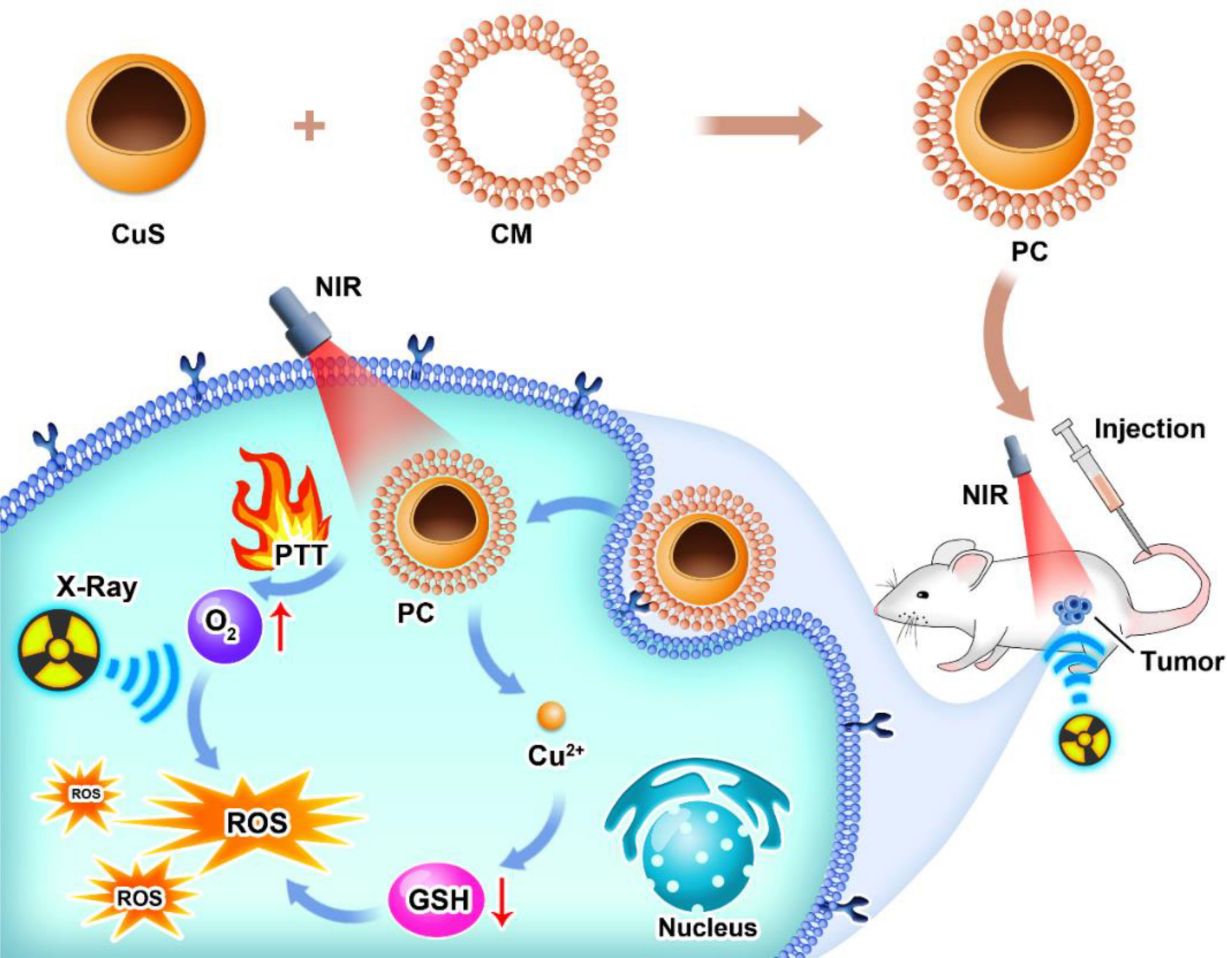
Illustration of PC preparation and cancer treatment.

**Figure 1:**
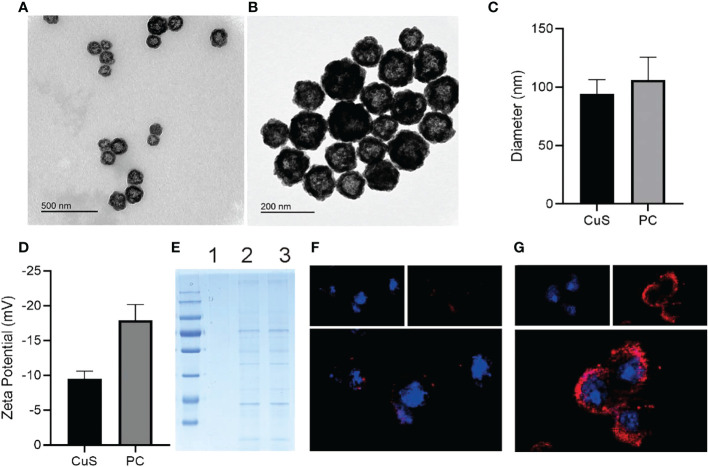
TEM images of CuS **(A)** and PC **(B)**. Zeta diameter **(C)** and potentials **(D)** of CuS and PC. SDS-pages of CuS (1), platelet cell membrane (2) and PC (3) **(E)**. CLSM images of CT 26 incubated with CuS **(F)** and PC **(G)**; cell nuclei was stained with DAPI and CuS, while PC was stained with DiL.

**Figure 2:**
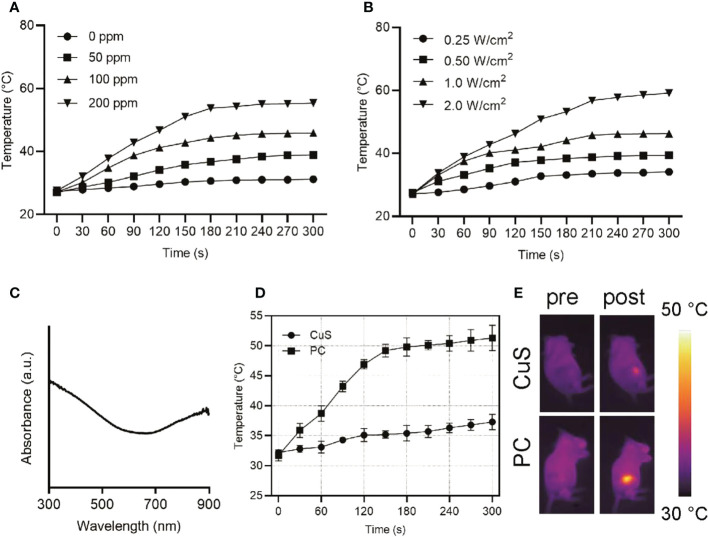
Heating curves of varying concentrations of PC upon 808 nm laser exposure at a power density of 1 W/cm^2^ **(A)**. Heating curves of varying power density at a PC concentration of 100 ppm upon 808 nm laser exposure **(B)**. UV-vis-NIR absorption spectra of PC at a concentration of 100 ppm **(C)**. Temperature change curves of tumors of mice administrated with CuS or PC after exposure to 808 nm laser at 1.5 W/cm^2^ **(D)** and IR images **(E)**.

As depicted in **Scheme 1**, we synthesized CuS NPs in two straightforward steps. Then, a platelet cell membrane (CM) was extracted from the platelets. Next, CuS was coated with CM to produce a composite nano-system coated with CM (PC). TEM analysis revealed the morphology and size distribution of CuS and PC nano-systems, revealing the spherical morphology of CuS and PCS with a size of approximately 100 nm (**Figures 1A, B**). Also, the average hydrodynamic size of CuS and PC was measured to be 94.1 nm and 106.0 nm, respectively, using dynamic light scattering (DLS) (**Figure 2D**), confirming the TEM results. Furthermore, PC was slightly larger than CuS, indicating that NPs were successfully encapsulated in the platelet cell membrane (**Figure 1C**). Furthermore, the zeta potential values of CuS and PCNPs were determined to be −9.5 ± 1.1 mV and −17.9 ± 2.3 mV (**Figure 1D**), respectively, which was attributed to CM encapsulation. Moreover, PC retained significant quantities of CM protein (**Figure 1E**), confirming the presence of functional membrane proteins in PC. The fluorescence images of CT 26 cells treated with CuS and PC are displayed in **Figures 1F, G**. The fluorescence of CuS and PC was Dil red, and the fluorescence intensity of PC was significantly greater than that of CuS. It indicates that the CuS-coated cell membrane was more readily recognized by tumor cells, thereby facilitating the targeting and accumulation of the PC composite system in tumor cells. Meanwhile, PC showed ability to depleting GSH under acidic condition (pH = 4.5) as shown in **Figure S1**.

UV-vis near-infrared spectroscopy of PC revealed high absorption at 808 nm (**Figure 2C**), indicating that PC has great application potential in NIR-I (650-1000 nm) laser photothermal conversion. Different concentrations of PC water dispersions were exposed to a 1 W/cm^2^ NIR-I laser for 5 minutes to investigate its photothermal conversion performance. A temperature rise profile dependent on concentration and time was observed (**Figure 2A**). After 5 minutes of NIR-I laser irradiation, the temperature of an aqueous solution containing 200 ppm of PC increased from 27.5°C to 55.4°C, significantly higher than the critical temperature of 42°C for tumor cell death. In addition, **Figure 2B** demonstrates that the temperature increase of a PC aqueous solution can be controlled between 34.1°C (0.5 W/cm^2^) and 59.1°C (1.5 W/cm^2^) by adjusting the applied power density of the NIR-I laser, demonstrating that the photothermal-transformation performance is highly dependent on the laser power density. In additional, PC exhibited remarkable photothermal stability after several heating up loops (**Figure S2**). Consequently, these results indicated that PC acted as a potential photothermal agent for its hyperthermia effect, owing to its excellent performance in promoting the efficient conversion of NIR-I laser light into thermal energy. Subsequently, we examined the *in vivo* photothermal conversion capability of PBS solution containing the same dose of CuS and PC when administered intravenously (**Figure 2D**). The temperature at the tumor site of mice in the PC group increased from 32.2°C to 51.3°C with extended irradiation time. In contrast, the temperature at the tumor site of mice in the CuS group increased by only 5°C, as can be seen intuitively from the IR image of mice in **Figure 2D** (**Figure 2E**). It suggests that the cell membrane will facilitate nanomaterials targeting the tumor site in mice. After laser irradiation, it will be easier to increase the temperature of the tumor site in order to ablate the tumor. Meanwhile, the hypoxia staining in **Figure S3** suggested the oxygen level elevation after laser irradiation.

**Figure 3:**
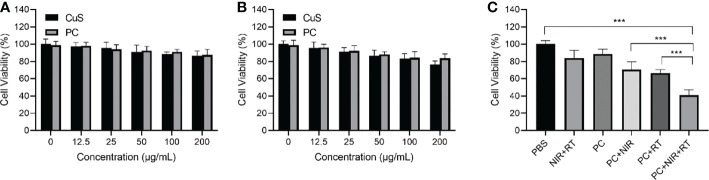
Cell viability of CT 26 cells **(A)** and FHC cells **(B)** after incubation with various doses of CuS and PC. Cell viability after treatment including control, NIR+RT, PC only, PC+NIR, PC+RT, and PC+NIR+RT **(C)**. ***p < 0.001.

PC biocompatibility was first evaluated to investigate PC’s radiosensitization effect *in vitro*. **Figures 3A, B** show that CT 26 and FHC cells exhibited satisfactory cell viability after incubation with CuS and PC, exceeding 70% cell viability at 200 µg/mL. The findings indicate that both CuS and PC are biosafe. Informed by the outcome, we analyzed the cell viability of CE 26 cells that had been subjected to a variety of treatments. The PC+NIR+RT group demonstrated a remarkable cancer cell-killing effect compared to the PBS group, but the survival rate was only 41.0% (**Figure 3C**). Notably, the therapeutic effect of the PC+NIR+RT group is superior to that of either the PC+NIR+RT group or the PC+RT group. This superiority can be attributed to the combined effect of PC-mediated photothermal therapy and radiotherapy. These *in vitro* findings indicate that PC can mediate photothermal therapy and radiotherapy and that PC-mediated thermoradiotherapy has a superior effect on inhibiting tumor cell growth.

**Figure 4:**
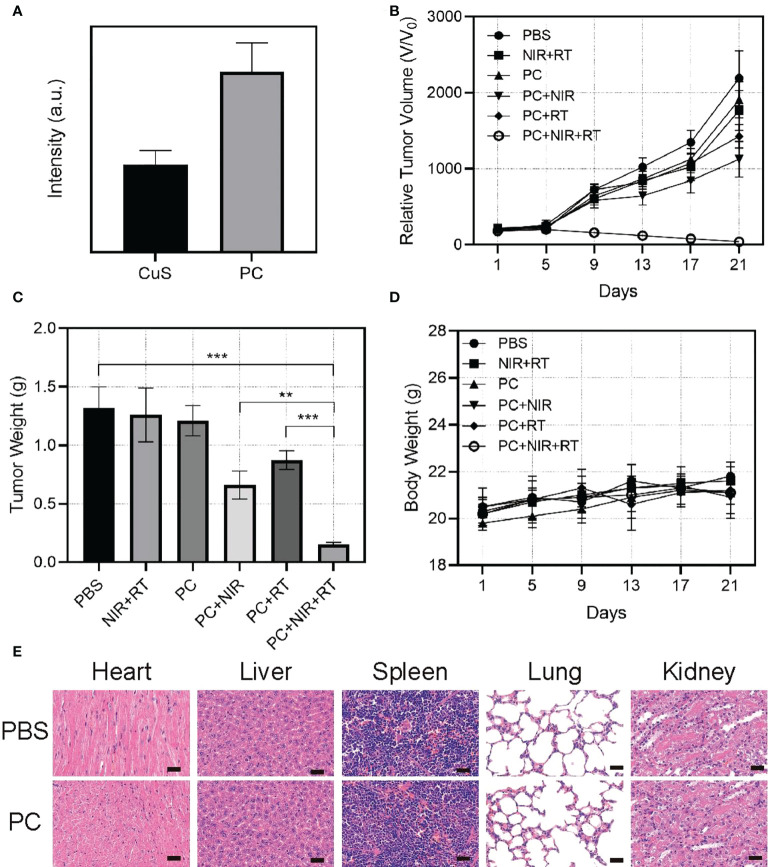
Fluorescence intensity of DiL in tumor tissue 24 hours after injection of DiL-labeled nanoparticles **(A)**. Relative tumor volume change **(B)**, and tumor weight **(C)** and body change **(D)**. Results of H&E-stained images for mice’s major organs, including the heart, lung, liver, kidneys, and spleen, after exposure to various therapies 21 days post-injection **(E)**. Scale bars: 75 µm. **p < 0.01, ***p < 0.001.

Given its excellent antitumor activity and other promising properties *in vitro*, we sought to assess PC’s capacity to mediate thermal radiation-induced tumor death *in vivo*. Before *in vivo* tumor therapy, we investigated the ability of PC to target tumors (**Figure 4A**). After CuS NPs and PC were injected intravenously into mice, the animals were sacrificed, and their tumors were collected for DiL (red) fluorescence imaging and intensity analysis. There were six distinct groups of mice corresponding to the type of treatment: 1) control group (PBS), 2) 808 nm near-infrared laser irradiation + radiotherapy (NIR+RT) (3), PC (4), PC+NIR (5), PC+ RT, and (6) PC+NIR+RT. To monitor treatment effects, we measured the average tumor size of mice in each treatment group over 22 days (**Figure 4B**). Compared to PBS-treated mice, RT and NIR alone were only associated with a partial delay in tumor growth. It suggests that NIR and RT alone are insufficient in this model system to affect tumor growth kinetics significantly. In contrast, PC was associated with a certain degree of tumor growth inhibition in the PC+NIR and PC+RT groups, consistent with the nanoparticles’ use as radiosensitizing and photothermal agents. Importantly, tumor growth was effectively inhibited in mice treated with PC+RT+NIR, confirming the ability of PC to ablate tumor tissue in the context of thermal radiation therapy. These results corresponded to measurements of tumor weight in these mice (**Figure 4C**), and none of these treatments were associated with any significant changes in mouse body weight (**Figure 4D**). **Figure 4E** demonstrates that the activation of the PC does not result in any system loss. The mice’s vital organs (heart, liver, spleen, lungs, and kidneys) were treated without causing inflammation or body damage. The *in vivo* data demonstrate that the designed PC system has effective antitumor properties due to its superior tumor targeting properties and the combined effect of PC-mediated photothermal therapy and radiotherapy.

## Conclusion

In this study, a platelet cell membrane-masked hollow CuS was successfully designed and fabricated. The as-prepared PC could efficiently navigate the tumor region without harming normal organs and tissues with platelet-resembling properties. Under acidic tumor microenvironment conditions, PC nanoparticles release quantities of Cu^2+^. Next, these Cu^2+^ ions consume intracellular GSH and thereby prevent potential OH scavenging. This process prevent ionization-damaged cancer cells from repairing themselves. In addition, photothermal therapy mediated by PC caused cancer cell death through mild hyperthermia. In addition, the local temperature increase in tumors contributed to the acceleration of intratumor blood flow, which aided in delivering more oxygen to tumors, thereby alleviating hypoxia. On the one hand, sufficient oxygen repairs radiotherapy-induced DNA double-strand breaks. On the contrary, GSH, which repairs cancer cell damage, was eliminated. Radioresistance was defeated *in vivo* and *in vitro* with the aid of PC. This research produced a novel PC strategy that could be applied to enhance the ablation of tumors and inhibit the self-repair of cancer cells.

## Data availability statement

The original contributions presented in the study are included in the article/**Supplementary Material**. Further inquiries can be directed to the corresponding authors.

## Ethics statement

The animal study was reviewed and approved by Jinan University.

## Author contributions

Conceived and designed the experiments: ZC, ML, and XL. Performed the experiments: LC, BW, and XZ. Contributed reagents/materials/analysis tools: ZC, Revised the polished the article: ML. All authors contributed to the article and approved the submitted version.

## Acknowledgments

The authors would like to thank Dr. Yufei Chen from Shiyanjia Lab (www.shiyanjia.com) for drawing schematic diagrams.

## Conflict of interest

The authors declare that the research was conducted in the absence of any commercial or financial relationships that could be construed as a potential conflict of interest.

## Publisher’s note

All claims expressed in this article are solely those of the authors and do not necessarily represent those of their affiliated organizations, or those of the publisher, the editors and the reviewers. Any product that may be evaluated in this article, or claim that may be made by its manufacturer, is not guaranteed or endorsed by the publisher.
