# Supplementary material for: Biomimetic CuS nanoparticles for radiosensitization with mild photothermal therapy and GSH-depletion
Source: Front Oncol. 2022 Nov 24;12:1054608. doi: 10.3389/fonc.2022.1054608 (PMC9730236; doi:10.3389/fonc.2022.1054608)
Supplement: Supplementary file 1 [file DataSheet_1.docx]

*Supporting information for*

**Biomimetic CuS nanoparticles for radiosensitization with mild photothermal therapy and GSH-depletion**

Xiaoxiang Zhou^1#^, Xiang Li^2#^, Bo Wu^1^, Zhiran Chen^1*^ and Longyun Chen^1*^

1.The Yancheng School of Clinical Medicine of Nanjing Medical University, Yancheng Third People’s Hospital

2. Department of Central Laboratory and Precision Medicine Center，

Department of Nephrology, The Affiliated Huai'an Hospital of Xuzhou Medical University and Huai’an Second People's Hospital, Huai'an, 223001, China.

#Author contributed equality

*Corresponding authors: Zhiran Chen, E-mail: [kk18751426705@163.com](mailto:kk18751426705@163.com) ;

Longyun Chen, E-mail: [chenlongyun@yeah.net](mailto:chenlongyun@yeah.net)

* X X Z and X L contributed equally to this work.

**
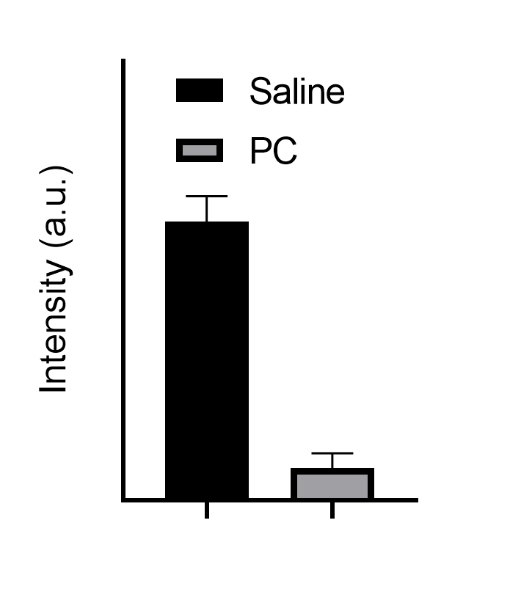
**

**Figure S1.** GSH content after co-incubation with saline or PC.

**
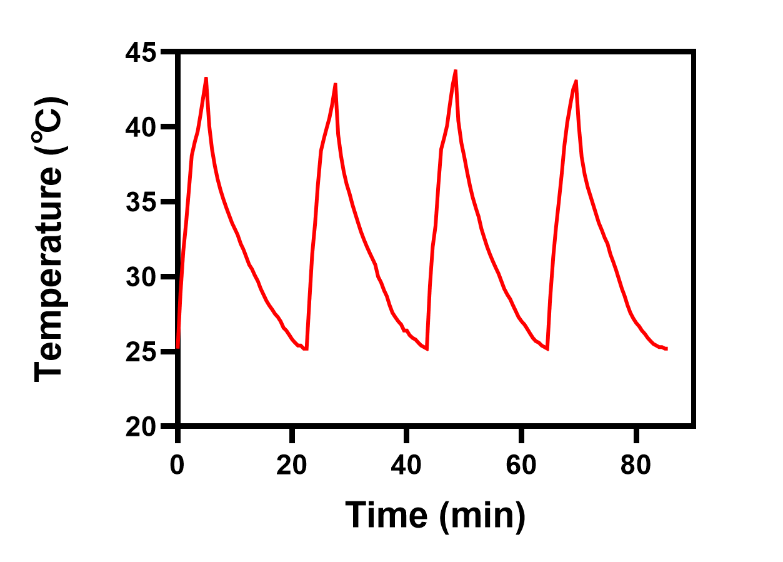
**

**Figure S2.** Photothermal cycle loops of PC.

**
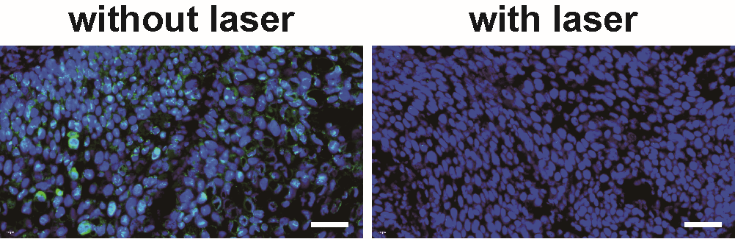
**

**Figure S3.** Pharmacokinetics study

**Experimental Section**

**Preparation of CuS and PC nanoparticles:** CuS nanoparticles were prepared using a previously reported method (Liu, W., et al. 2021). Briefly, a CuCl_2_ solution (100 μL) and poly(vinylpyrrolidone) (0.24 g) were mixed in 25 mL deionized water with continuously stirring at 25 ℃. Then 25 mL solution NaOH at pH 9 and 6.4 μL of hydrazine solution were added to the solution to obtain Cu_2_O spheres suspension, followed by Na_2_S (320 mg mL^−1^, 200 μL) solution added to the Cu_2_O suspension and reacted at 60 °C for 2 h. Next the mixture was centrifuged at 11 000 rpm for 10 min, followed by washing with deionized water twice and dried in vacuum to obtain hollow CuS nanoparticles.

To prepare PC, a previous reported method was conducted (Lyu, M., et al. 2021). Briefly, platelets derived from whole blood were collected via gradient centrifugation by first spinning 10 mL of whole murine blood at 1000 rpm for 20 minutes. The supernatant was then spun at 8000 rpm for 20 minutes. Then they were then repeatedly washed with PBS. The membrane vesicles and CuS were extruded through a 200 nm nuclepore polyester membrane on in a mini-extruder. The resultant particles were then spun for 10 minutes min at 10000 rpm to remove remaining platelet cell-derived vesicles and the resultant PC were collected and stored at 4 °C in PBS for subsequent experiments.

**Characterization of CuS and PC nanoparticles:** The morphology of CuS nanoparticles and PC nanoparticles were observed by transmission electron microscopy (TEM; Tecnai G2 F20 S-Twin, FEI, USA) at 100 keV acceleration voltage. The zeta potential and zeta diameter of the CuS NPs and PC NPs in 1 × PBS suspension were measured by dynamic light scattering (DLS, Nano-Zen 3600, Malvern Instruments, UK), respectively. The optical absorbance of PC NPs in the wavelength range of 300-800nm was measured effectively by UV-Vis near-infrared spectrophotometer (CARY5000, Varian Ltd., USA).

**Photothermal performance test:** PC NPs in water with different concentrations (0, 50, 100, 200 μg/ml) were irradiated with 808 nm laser (Changchun New Industries Tech.Co., Ltd, China) using a power density of 1 W/cm^2^ for 10 min. Then PC NPs at 100 μg/mL were irradiated with 808 nm laser at various power densities (0, 0.25, 0.5, 1, 2 W/cm^2^). After irradiation, turn off the laser and continue to record the temperature and time of natural cooling of the PC NPs. The data above were recorded every 10 s by an infrared thermal imager.

**Cell culture:** mouse colon cancer cell line (CT 26) and Fetal Human Colon cell line (FHC) were obtained from the Cell Bank of the Chinese Academy of Sciences, among them, CT 26 was incubated in RPMI-1640 medium supplemented with 10% FBS and FHC was incubated in DMEM medium supplemented with 10 % FBS, they were both incubated in an incubator containing 5% CO_2_ at an ambient humidity of 37 °C.

**Cytotoxicity assay (CCK-8):** we used the CCK-8 kit to evaluate the dark toxicity of different concentrations (0, 12.5, 25, 50, 100, 200 μg/mL) of PC and CuS NPs to cells. Firstly, CT26 cells and FHC cells were seeded separately into 96-well plates with a density of 5×10^3^ cells/well. Each type of cell was divided into 6 groups with 5 wells in each group and incubated for 24h. Then, the 6 groups were incubated with PC and CuS NPs at different concentrations (0, 12.5, 25, 50, 100, 200 μg/mL) for 24h, respectively. then 10 μL CCK-8 reagent was added to each well. After incubation for 2 h, the absorbance value at the characteristic peak at 450 nm was measured by a microplate analyzer (Rayto-6000 system, Rayto, China). The following we tested the effect of PC NPs (100 μg/mL) on cell viability. CT26 cells seeded into 96-well plates at a density of 5×10^3^ cells per well and were divided into 6 groups (5 Wells per group): (1) Control, (2) NIR+RT, (3) PC, (4) PC+NIR, (5) PC+RT, (6) PC+NIR+RT. Among them, the radiotherapy dose was 6Gy, and the concentration of PC was 100 μg/mL. The power intensity of the NIR laser is 1 W/cm^2^, irradiated for 5 minutes. The following operations are the same as described in the dark toxicity test.

**γ-H_2_AX immunofluorescence analysis:** CT26 cells were incubated at 37 °C for 24 h in six groups: (1) PBS injection, (2) NIR+RT (1 W/cm^2^, 5 min, 6 Gy), (3) PC (100 μL, 100 μg/mL), (4) PC+NIR, (5) PC+RT, (6) PC+NIR+RT PBS. After incubation for 24h, we first added 100μg/mL PC NPs to groups 3-6, followed by 4h incubation, irradiated groups 2,4 and 6 with 1 W/cm^2^ power intensity near-infrared light for 5 minutes, and finally irradiated groups 2,5 and 6 with 6Gy radiation dose. the cells were incubated at 37℃ for 4h. After three-times rinse with PBS, cells were fixed in 4 % paraformaldehyde for 20 min, treated by 0.1 % Triton-X-100 and later blocked at room temperature for 2 h. Washed with PBS for three times, the cells were dyed with DAPI and secondary anti-γ H_2_AX antibody with 5 % FBS and 1% Trition-X-100 before washed with PBS. The cells immunofluorescence was analyzed by a fluorescent microscope (IX81, Olympus, Japan).

**Fluorescence analysis of intracellular ROS:** CT26 cells were incubated in a six-well plate at 37℃ for 24 h and divided into 6 groups: (1) Control, (2) NIR+RT, (3) PC, (4) PC+NIR, (5) PC+RT, (6) PC+NIR+RT. After incubation for 24 h, we first added 100 μg/mL PC NPs to groups 3-6, followed by 4h incubation, irradiated groups 2,4 and 6 with 1 W/cm^2^ power intensity near-infrared light for 5 minutes, and finally irradiated groups 2,5 and 6 with 6Gy radiation dose. the cells were incubated at 37℃ for 4h. The old culture medium was removed, diluted DCFH-DA was added to the six-well plate, and the cells were cultured at 37℃ for 20 min. The cells were washed with serum-free culture medium for three times, and then the ROS fluorescence level was directly observed using a Japanese laser confocal microscope.

***In Vitro* Cancer Targeting Study:** CuS and DiL were first co-stirred to label DiL (the marking method for PC NPs is the same), and then CuS and PC labeled separately with DiL were co-incubated with CT 26 cells for 4 h at 37 °C. The cells were then washed with PBS several times, fixed with PFA for 30 min at room temperature, stained with DAPI and then imaged by using a confocal laser scanning microscope (CLSM; IX81, Olympus, Japan).

**Colony formation assay:** FHC cells were seeded into 6-well plates with 500 per well and incubated at 37 °C for 24 h. The radiation dose curve experiments were designed into three groups with 6 complex wells in each group: (1) PBS, (2) PC, (3) PC+NIR. After 4h of incubation with PC NPs suspension (100μg/mL), three washings were performed. Irradiated groups 3 with 1 W/cm^2^ power intensity near-infrared light for 5 minutes. Then 6 six-well plates were set up for each treatment, and different radiotherapy doses (0, 2, 4, 6, 8 Gy) were performed respectively. 10 days later, cells were stained with Crystal violet dye. This process calls the standard linear quadratic model, counts the colonies covering at least 50 cells, and completes the effective calculation of the colony formation rate. Then, the survival rate of the colony was calculated to realize the evaluation of the effects of various treatments.

**Animal models：**4 to 5-week-old female BALB/c mice (purchased from Vital River Company, Beijing, China) were subcutaneously injected with 100 μL CT26 cell suspension (1×10^7^ cells/mL) on the right side of axillary to establish tumor model.

***In vivo* infrared thermography:** To monitor the *in vivo* photothermal effect, 100 μL CuS and PC suspension with 100μg/mL concentration were injected into the tail vein of the tumor-bearing mice, respectively. And then the tumors were irradiated by 1.5 W/cm^2^ laser irradiation for 5 min at 1 h post-injection. PBS injection used as control group. Meanwhile, the temperature at the tumor was monitored using an infrared camera (Fotric 225) every 30 seconds.

***In vivo* antitumor study:** When tumor size reached approximately 200 mm^3^, the mice were divided randomly into 6 groups (each group included 5 mice): (1) PBS injection, (2) NIR+RT (1.5 W/cm^2^, 5 min, 6 Gy), (3) PC (100 μL, 100 μg/mL), (4) PC+NIR, (5) PC+RT, (6) PC+NIR+RT PBS. The concentration of NPs, the intensity and duration of NIR irradiation and the dose of radiotherapy were all the same in each group. The day of treatment was recorded as the first day, and the length and width of the tumors and weight of the mice were recorded with calipers and weight scales every 3 days until the end of the 22-day treatment, the mice were euthanized and their tumors were taken and weighed. the tumor volume was calculated according to following formula. Tumor volume = tumor length × tumor width^2^ / 2.

**Immunofluorescent staining:** After 22 days treatment, all the mice were sacrificed. Five main organs (heart, liver, spleen, lung and kidney) of all mice were harvested, washed with PBS, and fixed with paraformaldehyde for histology analysis. And the tumor tissues were weighed, and fixed in 4% neutral buffered formalin, processed routinely into paraffin, and sectioned at 4μm. Then the sections were stained with HIF-1α, Ki-67 and terminal deoxynucleotidyl (TUNEL) staining and finally examined by using an optical microscope (BX51, Olympus, Japan).

**HE staining:** Mice (n=3) with tumor volume of 200 mm^3^ were injected 100uL PBS and PC suspension with 100μg/mL concentration by tail vein, respectively, and vital organs including hearts, livers, spleens, lungs and kidneys were harvested 14 days after treatment. Then fixed in 4% formaldehyde and embedded in paraffin. The paraffin was sliced at stained with hematoxylin and eosin (H&E).

**Biodistribution study:** The CT26 tumor bearing BALB/c mice (n = 3) were divided into two groups when the tumor was 200mm^3^. 100uL CuS and PC suspension with 100μg/mL concentration were injected into the tail vein, respectively. All mice were euthanized 24h later, and their major organs (heart, liver, spleen, lung, kidney) and tumors were collected and aqua regia was added to them. The mixture was left at room temperature for 12 h, followed by annealing at 70 °C for 6 h to remove the acids. The sample was then re-suspended with 1 mL DI water and the Cu content in each sample was determined by using an inductively coupled plasma-atomic emission spectrometer (ICP-AES; iris Intrepid II XSP, Thermo Elemental, USA).

Liu W., Xiang H., Tan M., Chen Q., Jiang Q., Yang L., Cao Y., Wang Z., Ran H. and Chen Y.: Nanomedicine Enables Drug-Potency Activation with Tumor Sensitivity and Hyperthermia Synergy in the Second Near-Infrared Biowindow. *ACS Nano*, 15(4), 6457-6470 (2021) doi:10.1021/acsnano.0c08848

Lyu M., Chen M., Liu L., Zhu D., Wu X., Li Y., Rao L. and Bao Z.: A platelet-mimicking theranostic platform for cancer interstitial brachytherapy. *Theranostics*, 11(15), 7589-7599 (2021) doi:10.7150/thno.61259
